# High-Q Trampoline Resonators from Strained Crystalline InGaP for Integrated Free-Space Optomechanics

**DOI:** 10.1021/acs.nanolett.3c00996

**Published:** 2023-05-26

**Authors:** Sushanth Kini Manjeshwar, Anastasiia Ciers, Fia Hellman, Jürgen Bläsing, André Strittmatter, Witlef Wieczorek

**Affiliations:** † Department of Microtechnology and Nanoscience (MC2), Chalmers University of Technology, SE-412 96 Gothenburg, Sweden; ‡ Institute of Physics, Otto von Guericke Universität Magdeburg, 39106 Magdeburg, Germany

**Keywords:** nanomechanics, optomechanics, photonic crystal, radiation loss, high stress, InGaP

## Abstract

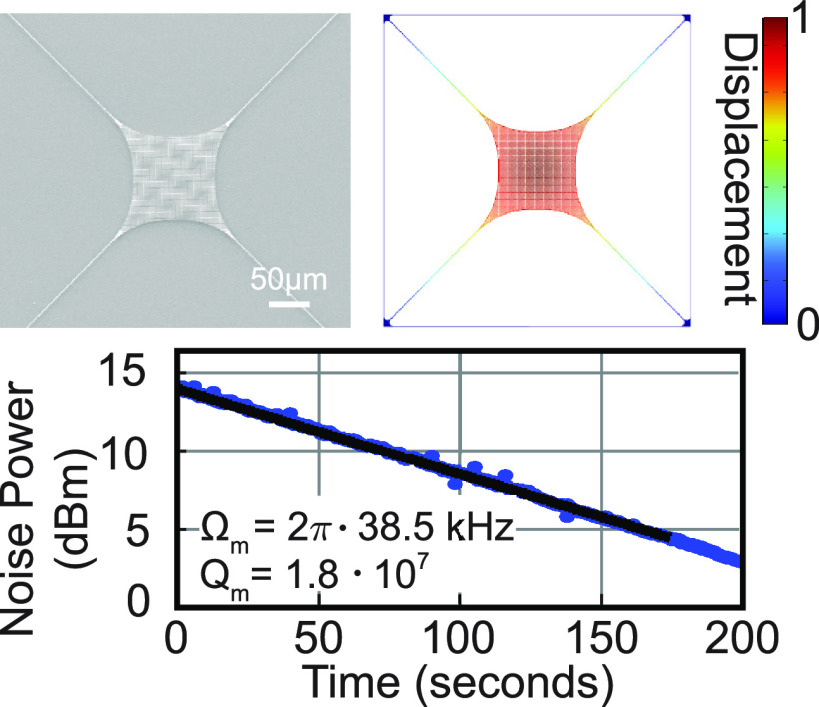

Nanomechanical resonators realized from tensile-strained materials reach ultralow mechanical dissipation in the kHz to MHz frequency range. Tensile-strained crystalline materials that are compatible with epitaxial growth of heterostructures would thereby at the same time allow realizing monolithic free-space optomechanical devices, which benefit from stability, ultrasmall mode volumes, and scalability. In our work, we demonstrate nanomechanical string and trampoline resonators made from tensile-strained InGaP, which is a crystalline material that is epitaxially grown on an AlGaAs heterostructure. We characterize the mechanical properties of suspended InGaP nanostrings, such as anisotropic stress, yield strength, and intrinsic quality factor. We find that the latter degrades over time. We reach mechanical quality factors surpassing 10^7^ at room temperature with a *Q*·*f* product as high as 7 × 10^11^Hz with trampoline-shaped resonators. The trampoline is patterned with a photonic crystal to engineer its out-of-plane reflectivity, desired for efficient signal transduction of mechanical motion to light.

Mechanical dissipation in nano- and micromechanical resonators has been drastically reduced in recent years by the use of dissipation dilution, soft clamping, and strain-engineering techniques.^1−6^ Most of these methods require the use of tensile-strained materials, such as the widely employed amorphous SiN^4,5,7−12^ and, more recently, crystalline materials such as SiC,^13,14^ Si,^15^ GaNAs,^16^ and InGaP.^17−19^ Ultrahigh-quality-factor mechanical resonators fabricated from these materials open up exciting prospects for nanomechanical sensing by reaching unprecedented force sensitivities^15,20,21^ and, when the resonators are coupled to light, pave the way for generating optomechanical quantum states at room temperature.^8,22,23^ Strained crystalline materials compatible with epitaxial layer growth can realize integrated cavity optomechanical devices through bottom-up growth and top-down microfabrication. At the same time, this integrated approach would enable on-chip stability and scalability. Current optomechanical devices incorporating chip-based mechanical resonators that are coupled to out-of-plane light resort to stacking of multiple chips^24^ or to assembling independent components.^25,26^ Integrating the free-space optical cavity and the mechanical resonator on a single chip would provide alignment-free devices with ultrasmall mode volumes to drastically increase the interaction strength between out-of-plane light and mechanical motion.

InGaP is a crystalline material that can be epitaxially grown on (Al,Ga)As and, therefore, would enable realization of integrated free-space cavity optomechanics on a chip. Further, InGaP can be grown with tensile strain on (Al,Ga)As determined by the Ga content of the InGaP layer and thus has the potential to lead to ultralow dissipation mechanical resonators. Tensile-strained nanomechanical resonators fabricated from InGaP have been recently demonstrated in membrane^17^ and string-type geometries.^18,19^ Membrane-type resonators have thereby reached quality factors of up to 10^6^ at room temperature.^17^ Further, it was experimentally confirmed that stress is anisotropic in InGaP,^18^ which opens up new avenues for strain engineering the geometry of nanomechanical resonators.

In our work, we demonstrate trampoline-shaped nanomechanical InGaP resonators that combine low mechanical dissipation with engineered optical reflectivity, a crucial step toward free-space cavity optomechanics on a chip. We achieve mechanical quality factors surpassing 10^7^ at room temperature with trampoline-shaped nanomechanical resonators, which employ a simple geometry to dilute the material’s intrinsic dissipation.^8,9,14,27^ For transduction of mechanical displacement to the light field we engineer the out-of-plane reflectivity of the resonator at telecom wavelengths by patterning the central area of the 73 nm thick InGaP trampoline with a photonic crystal.^8,26,28−31^ We first study the mechanical material properties of the strained InGaP layer^17−19^ by fabricating and characterizing string resonators to determine the intrinsic stress, yield strength, and the intrinsic quality factor. We then demonstrate high-*Q* trampoline-shaped InGaP nanomechanical resonators with engineered optical reflectivity.

We fabricate InGaP string- and trampoline-shaped mechanical resonators from a III–V material heterostructure grown via metal–organic chemical vapor deposition (MOCVD). A 400 nm thick GaAs buffer layer is grown on a GaAs substrate along the [0 0 1] crystal direction followed by a 73 nm thick In_1–*x*_Ga_*x*_P layer, a sacrificial layer of Al_0.68_Ga_0.32_As with a thickness of 785 nm, and another In_1–*x*_Ga_*x*_P layer of 75 nm thickness. Note that the as-grown structure would allow for implementing sub-μm-spaced two-element optomechanics on a chip.^26^

The devices in this work were fabricated after stripping the top In_1–*x*_Ga_*x*_P and Al_0.68_Ga_0.32_As layers. From X-ray diffraction analysis we find that the gallium content in the as-grown InGaP layer is 0.53 ≤ *x* ≤ 0.59, with a value of *x* = 0.5658 matching our inference of released stress from InGaP string resonators. Hence, we will assume throughout the rest of this work *x* = 0.5658 (abbreviated as 0.57). We use electron-beam lithography to expose the resonator patterns to a resist stack containing an adhesion promoter and an electron beam resist. Chlorine-based RIE-ICP etching is then used to transfer the pattern onto the InGaP device layer followed by releasing the devices with a selective anisotropic wet etch using a mixture of citric acid and hydrogen peroxide.^32^ Finally, we perform critical point drying to prevent the devices from collapsing due to capillary forces. Figure 1 shows fabricated trampoline resonators.

**Figure 1:**
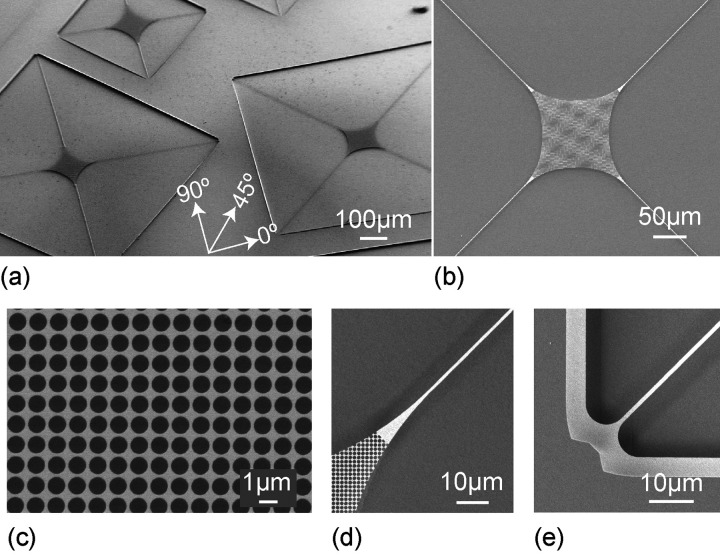
Tensile-strained trampoline resonators made from a 73 nm thick crystalline InGaP layer. Scanning electron microscope (SEM) images of (a) trampoline resonators oriented along different crystal directions and varied tether length (tether width 1 μm, central pad area 100 × 100 μm^2^). The angles 0°, 45°, and 90° denote the crystal directions [1 1 0], [1 0 0], and  respectively. (b) Close-up of a trampoline resonator with a tether length of 750 μm. Enlarged views of the resonator in (b) showing (c) the photonic crystal (PhC) pattern on the central area with PhC hole radius *r*_PhC_ = 544 nm and period *a*_PhC_ = 1304 nm, (d) the tether connection to the central pad, and (e) the tether clamping to the substrate.

Material properties of the InGaP layer, in particular its tensile stress, yield strength, and intrinsic mechanical quality factor, are key factors to engineer high-quality mechanical resonators at desired eigenfrequencies. We determine these material properties experimentally by fabricating nanostring resonators, following methods introduced in refs (18), (19), (33), and (34).

The intrinsic stress in the In_0.43_Ga_0.57_P layer originates from its lattice mismatch with the GaAs buffer layer. The in-plane as-grown strain of the thin In_1–*x*_Ga_*x*_P layer is
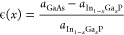
1where *a*_GaAs_ and  are the lattice constants of GaAs and In_1–*x*_Ga_*x*_P, respectively. One can tune the as-grown stress in the In_1–*x*_Ga_*x*_P device layer by varying *x* to obtain compressive stress for *x* < 0.515 and tensile stress for *x* > 0.515 (see Supporting Information). The InGaP layer can be grown without defects until a certain critical thickness governed by *x*, which is 1132 nm for *x* = 0.5658.^35^ In our case, the InGaP device layer has an as-grown thickness of 73 nm, which is well below this limit (see Supporting Information).

The crystalline structure of In_1–*x*_Ga_*x*_P results in an orientation-dependent released axial stress σ(*x*, θ)^18^ (for details see Supporting Information)
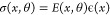
2where the angle θ is defined with respect to the crystal directions as shown in the inset of Figure 2. Importantly, σ(*x*, θ) is the released stress as Young’s modulus *E*(*x*, θ) accounts for an anisotropic Poisson ratio (see Supporting Information). We determine the anisotropic stress in In_0.43_Ga_0.57_P from measurements of string resonators of different lengths oriented along different directions. The eigenmode frequencies of tensile-strained string resonators are given by^36^
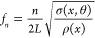
3where *n* is the mode number, ρ(*x*) is the density of the material, and *L* is the length of the resonator.

**Figure 2:**
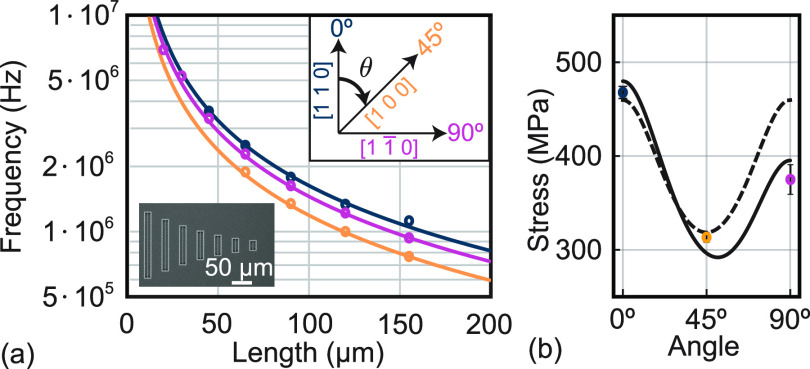
Tensile stress of the 73 nm thick In_0.43_Ga_0.57_P string resonators. (a) Mechanical frequencies of the fundamental mode of string resonators of different lengths (with a width of 200 nm) along the three crystal directions [1 1 0], [1 0 0], and  denoted as 0°, 45°, and 90°, respectively. The lines are a fit to the expected frequencies of tensile-strained string resonators (eq 3). The inset shows an optical microscope image of string resonators of different lengths oriented along 0°. (b) The extracted tensile stress along different crystal directions is shown as points. The dashed line shows the tensile stress σ(*x*, θ) predicted from in-plane strain ϵ(*x*) and Young’s modulus *E*(*x*, θ); see eq 2. The solid line shows the tensile stress that takes into account an additional angle-dependent contribution to Young’s modulus (see eq 4).

We fabricated string resonators with lengths between 20 and 160 μm and a width of 200 nm along the crystal directions [1 1 0], [1 0 0], and  We measured their thermally driven displacement noise power spectrum (NPS) in a high vacuum environment with an optical homodyne detection setup; for details see ref (31). The same setup was used for characterizing the mechanical properties of the InGaP trampoline resonators. Figure 2a shows the measured fundamental eigenmode frequencies of the 200 nm wide string resonators. We use the measured eigenfrequencies of all identified eigenmodes to determine the stress along the different crystal directions using eq 3 and ρ(0.57). We obtain a released stress in the string resonators of σ(0°) = 467.7(71) MPa, σ(45°) = 313.3(54) MPa, and σ(90°) = 374.9(164) MPa.

We can estimate the Ga content of the In_1–*x*_Ga_*x*_P layer based on the experimentally determined stress values by using eq 2. Note that this equation accounts for stress relaxation by incorporating Poisson’s ratio in *E*(*x*, θ) (see Supporting Information). We estimate a Ga content of 0.5667, 0.5649, and 0.5566 from the stress along 0°, 45°, and 90°, respectively. As the Ga contents along 0° and 45° are similar, we use the average value of *x* = 0.5658 to estimate the expected crystal-direction-dependent released stress, seen as the dashed-line in Figure 2b. This prediction captures the released stress along 0° and 45°, as expected, but not along 90°. However, from the crystal structure, we would expect the stress to be identical along the 0° and 90° directions. This unexpected deviation has also been observed in ref (18), which attributed it to a defect density that varies along different crystal directions. Alternatively, spontaneous ordering in MOCVD growth may also be a possible reason.^37^ This modification of Young’s modulus, Δ*E*(*x*, θ), can be modeled with a cos(2θ) function as
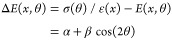
4

We obtain α = −5.9 GPa and β = 11.3 GPa (in ref (18) α = −5.5 GPa, β = 5.1 GPa). The stress including the deviation Δ*E*(*x*, θ) is shown as the solid line in Figure 2b and captures the data well. We attribute the remarkably small difference between our determined values for α and β and the ones from ref (18) to the difference in growth method (MOCVD vs MBE), the gallium content (0.5658 vs 0.59), and the resonator’s support geometry. Experimental determination of Young’s modulus along the crystal directions of InGaP^38^ together with detailed material studies is required to explore the microscopic origin for this additional anisotropy.

It is desirable to maximize the strain (respective stress) in the mechanical device layer to increase the effect of dissipation dilution. Since InGaP is a brittle material, a limit is set by the maximal applicable stress, i.e., σ_yield_(θ), after which the material breaks. We determine the yield strength of the InGaP layer experimentally following the method from ref (34). In our case, the yield stress depends on the orientation of the string resonators with respect to the crystal directions. We obtain yield stresses σ_yield_ of 5.5(8) GPa, 3.3(5) GPa, and 3.7(5) GPa along 0°, 45°, and 90°, respectively (see Supporting Information). The corresponding yield strain of ϵ_yield_(θ) = σ_yield_(θ)/[*E*(θ) + Δ*E*(θ)] is 0.043(8), 0.041(8), and 0.034(5), along 0°, 45°, and 90°, respectively. The obtained yield strength is comparable to Si_3_N_4_ (6 GPa) but lower than the one of SiC (21 GPa) or diamond (35 GPa).^6^

The quality factor *Q* of a mechanical resonator is generally given by^39^
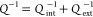
5where *Q*_int_ and *Q*_ext_ are the quality factors limited by intrinsic and extrinsic loss mechanisms, respectively. In the following, we determine *Q*_int_, which captures material-related loss processes of the 73 nm thick InGaP layer. To this end, we use string resonators and we confirmed that they are not limited by clamping loss or gas damping (Supporting Information), which determine *Q*_ext_ in our case.

We determine *Q*_int_ of the 73 nm InGaP layer from measurements of the quality factor of strained InGaP string resonators. Importantly, the stress in the string resonators dilutes *Q*_int_ by a factor *D*(6,33,36)
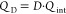
6The dilution factor *D* depends on the stress, resonator geometry and displacement mode profile. For a uniform string resonator, one obtains^33,40^
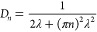
7where *n* is the mode number and λ is a stress parameter given as
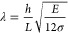
8with length *L* and thickness *h* of the string resonator.

We fabricated strings of different lengths with a width of 2 μm oriented along different crystal directions to infer *Q*_int_. Figure 3a shows the measured quality factors for the fundamental mode extracted from ringdown measurements. Using *D*_1_, we obtain *Q*_int_ of 7550 ± 140 and 8150 ± 320 along 0° and 90°, respectively. Our determined average *Q*_int_ of about 7.9 × 10^3^ for the 73 nm thick InGaP layer is comparable to LPCVD-grown Si_3_N_4_ (66 nm thick, *Q*_int_ =3.75 × 10^3^;^41^ with *Q*_int_ = 6.9 × 10^3^ × *h*[100 nm] ^33,41^ for 73 nm SiN, *Q*_int_ = 5 × 10^3^) and 14 nm thick s-Si (8 × 10^3^ ^15^) and larger than for 75 nm thick SiC (1.5 × 10^2^ ^14^).

**Figure 3:**
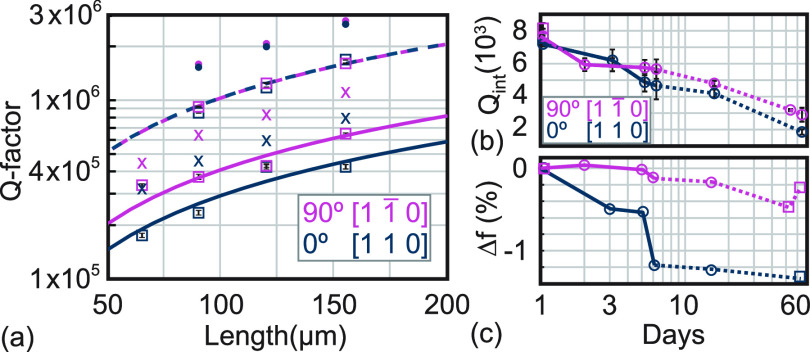
Determination of the intrinsic mechanical quality factor using InGaP string resonators. (a) Measured *Q* factors (squares) for the fundamental mode of string resonators with varying lengths oriented along 0° and 90°. The dashed (solid) lines are fits to extract *Q*_int_ at day 1 (day 60). The dots (crosses) show *Q*_D_^FEM^ obtained from FEM, when using *Q*_int_ from day 1 (day 60) as input. (b) We observe that *Q*_int_ degrades over time, shown for two samples (squares and circles). The solid (dashed) line indicates when the sample was stored in vacuum (ambient condition). (c) The relative change of the resonance frequency of the string resonators, i.e., Δ*f* = (*f*_day *x*_ – *f*_day 1_)/*f*_day 1_, is shown over the same time period.

The dilution factor can be numerically computed using FEM simulations (see Supporting Information).^27,33,42^ This approach is required when analyzing dissipation dilution of more complex mechanical resonator geometries, as in our case, trampoline mechanical resonators including a PhC pattern. We verify the FEM approach by simulating the dilution factor *D*_FEM_ for string resonators. Using the experimentally determined intrinsic quality factor, *Q*_int_, we calculate *Q*_D_^FEM^ = *D*_FEM_*Q*_int_, which is shown in Figure 3a. We find that *Q*_D_^FEM^ is slightly larger than the measured *Q* factors, similar to other works.^14,27,43^

We observe that the mechanical quality factor of the string resonators decreases with time. Figure 3b shows this change of *Q*_int_ for two different samples. The samples were measured in high vacuum, but they were stored in between measurements either in vacuum or under ambient conditions; see Figure 3b. *Q*_int_ decreases over two months by up to a factor of 4. During the same time period the resonance frequency changes by less than 2%; see Figure 3c. We hypothesize that the degradation of the quality factor is not due to a gradual relaxation of the tensile stress of the InGaP layer. At the moment, we can only speculate that the InGaP layer undergoes some modification, for example, moisture-induced degradation^44^ or other processes^45^ that may lead to an increase of mechanical dissipation. Future work is required to determine the cause of the InGaP degradation. To this end, the InGaP layer can be examined periodically with X-ray diffraction (to obtain information about the formation of an oxide surface layer) and photoemission spectroscopy (to obtain data on the elements present on the surface and their chemical state). Scanning near-field optical microscopy, micro-Raman, or tip-enhanced Raman spectroscopy can be used on the InGaP nanomechanical resonators to obtain, e.g., spatial information about potential strain changes. Mitigation strategies include surface passivation^46^ or capping of the InGaP layer with thin GaAs layers.

For efficient transduction of mechanical motion to out-of-plane light, the reflectivity of the mechanical resonator is desired to be close to unity. At the same time, the thickness of the device layer should be sufficiently thin to keep mechanical damping small. These requirements can be fulfilled by patterning thin mechanical resonators with a PhC.^8,26,28−31^ We, therefore, choose a trampoline-shaped geometry, which allows patterning its central area with a PhC to achieve the desired reflectivity and at the same time allows decreasing mechanical dissipation by use of dissipation dilution;^8,9^ see Figure 4b.

**Figure 4:**
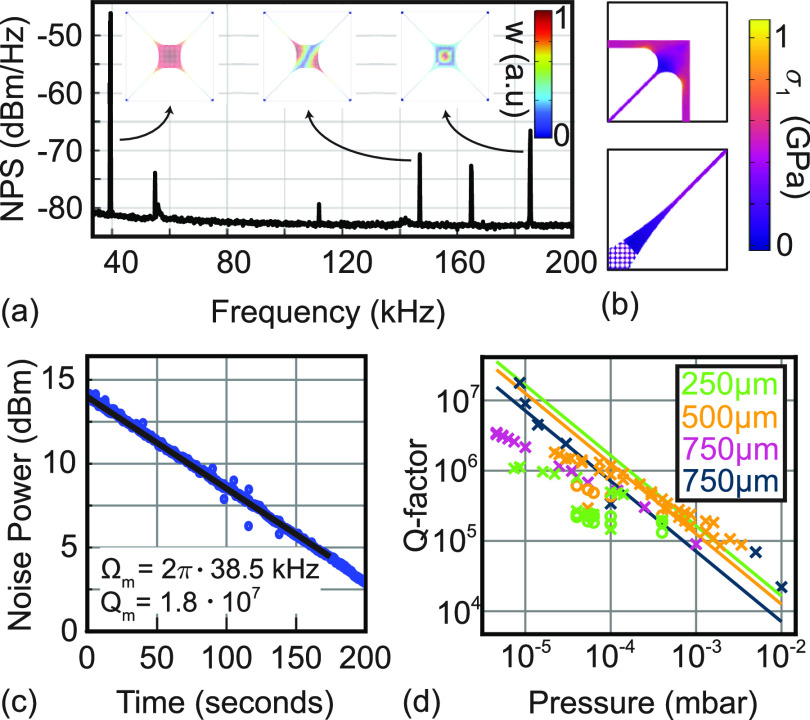
Mechanical properties of InGaP trampoline resonators. (a) Noise power spectrum (NPS) of a trampoline resonator of 750 μm tether length, 1 μm tether width, and central PhC pad size of 100 × 100 μm^2^. The insets show FEM simulated mode shapes depicting the out-of-plane displacement *w*. (b) FEM simulations of the first principal stress in the released device at the tether connection to the pad and the clamping region. (c) Ringdown measurement on day 5 of the fundamental mode of the trampoline from (a), which was placed in vacuum directly after fabrication on day one. We obtain a *Q* of 1.8 × 10^7^ from a fit (solid line) to the decay. (d) Dependence of *Q* on pressure for the fundamental mode of trampolines of different lengths with tethers oriented along different crystal directions (0°/90° marked by a circle, 45°/135° by a cross). The solid lines show the quality factor limited by gas damping for trampolines of tether lengths 250 μm, 500 μm, and 750 μm.

References (8) and (9) demonstrated that high-*Q* trampolines can be realized with thin and long tethers that connect the central pad to the support. In our work, we can reliably fabricate InGaP trampolines with a tether width of 1 μm and tether length of up to 750 μm (Figure 1b) and with a radius of 10 μm at the tether clamp to the support (Figure 1e) and of 200 μm at the tether clamp to the pad (Figure 1d).

Figure 4a shows a thermally driven displacement noise power spectrum of an InGaP trampoline resonator. The tethers of this device are 750 μm long and oriented along 45°/135°. We observe the fundamental mode at 38.5 kHz and several higher-order modes, which we identify by comparing measured eigenfrequencies to the ones simulated via FEM. Table 1 shows measured fundamental mode eigenfrequencies for trampolines with various tether lengths. As expected, we find that trampolines with shorter tether lengths exhibit higher resonance frequencies. We observe that trampolines whose tethers are oriented along 0°/90° have larger frequencies than the ones oriented along 45°/135°. We can understand this behavior as the stress along 0°/90° is larger than the one along 45°/135° (see Figure 2b) resulting in a higher resonance frequency. We find a good agreement with the eigenfrequencies calculated with FEM. In the FEM simulations, we take into account the anisotropy of Young’s modulus (eq 2) but do not account for its deviation (eq 4), which is a possible reason for the small discrepancy between the FEM and measurement results.

**Table 1:** Measured and FEM-Simulated Eigenfrequencies of the Fundamental Mode of Trampolines with Varied Tether Length and Orientation

tether		frequency (kHz)	
length (μm)	orientation	measured	simulated
250	0°/90°	90.9	106.9
	45°/135°	80	90.5
500	0°/90°	54.2	62.3
	45°/135°	43.7	52.4
750	0°/90°	40.1	47.8
	45°/135°	38.5	40.1

The highest mechanical *Q* factor that we measure is 1.8 × 10^7^ for the fundamental mode of the InGaP trampoline with 750 μm tether length (see Figure 4c), resulting in a *Q*·*f* product of 7 × 10^11^ Hz. We measured this value at room temperature at a pressure of 8 × 10^–6^ mbar, which is close to the minimal achievable pressure that we can reach in our setup. With the current devices, we reach a calculated thermal noise limited force sensitivity of 50 aN/ When compared to SiN-based membrane-type devices at room temperature, our value lies in the same order of magnitude as reached with phononic band gap SiN membranes (37 aN/ ^21^) and SiN trampolines (19.5 aN/ ^8,9^).

We already noticed that the mechanical *Q* of the InGaP string resonators decreases with time. We observe the same trend for the InGaP trampoline resonators. This behavior complicates a definite identification of the loss mechanism that limits the *Q* of InGaP trampolines. Nevertheless, we look at different mechanical damping mechanisms in the following to analyze limits in achieving even higher *Q*. We consider gas damping first. To this end, we performed pressure-dependent measurements of various trampolines; the results are shown in Figure 4d. We observe a linear increase of *Q* with a decrease in pressure, as expected from gas damping^36^ (Supporting Information). The *Q*-factor of the 750 μm long trampoline (dark blue crosses) that was fabricated and immediately measured follows this gas-damping prediction. However, for samples that were measured with a delay after fabrication (other colors in Figure 4d), we observe a deviation from the gas damping limit at lower pressures, which indicates that the *Q* factor of these trampolines reaches another limiting mechanism. As reaching low pressures requires some days of pumping, this deviation may originate from the degradation of *Q*_int_ over time. The amount of dissipation dilution achieved with the trampoline geometry may also limit the maximally achievable *Q*. To evaluate this, we computed *D*_*Q*_ via FEM and obtain a value of *D*_*Q*_ = 1750 for the 750 μm tethered trampoline. With *Q*_int_ = 7.9 × 10^3^ we obtain *Q*_*D*_ ∼ 1.38 × 10^7^. This *Q* factor is close to the experimentally obtained result. Hence, the trampoline resonators may currently be limited by the achievable gas pressure or by the amount of dissipation dilution. Stabilization of *Q*_int_ is required to identify with certainty the limiting damping mechanism and apply strategies to further reduce it.

In the following, we characterize the optical reflectance of the trampoline resonators patterned with a PhC. For details of the measurement setup, we refer the reader to ref (31). Figure 5a shows reflectance spectra of three trampolines with square PhC patterns of lattice constant *a*_PhC_ = 1309 nm and PhC radii *r*_PhC_ of 480 nm, 553 nm, and 605 nm. We evaluated the PhC parameters via image recognition applied to high-magnification SEM images of the respective PhC pattern after fabrication. We observe that the PhC trampolines demonstrate an engineered reflectance in the wavelength range of 1510–1620 nm with a pronounced modulation. The latter can be understood by noting that the trampoline is separated from the GaAs substrate by a vacuum gap of about 15 μm, originating from the release of the trampoline in the wet etch fabrication step. This gap forms a low-quality optical cavity between the trampoline and the GaAs substrate.

**Figure 5:**
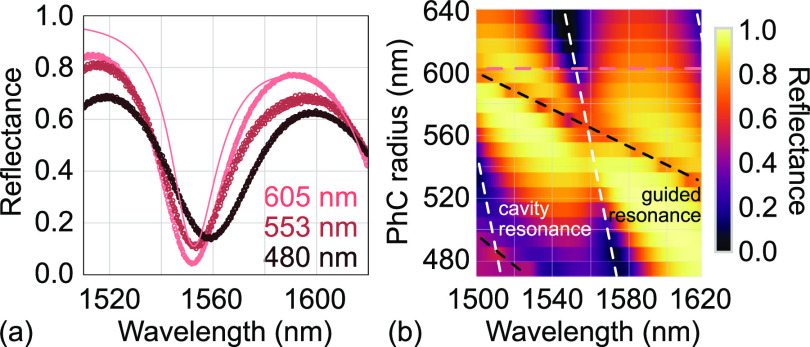
Reflectance spectra of InGaP trampoline resonators patterned with a PhC. (a) Measurements for *r*_PhC_ of 480 nm, 553 nm, and 605 nm (dots) and RCWA simulation for *r*_PhC_ = 605 nm (solid line). (b) Simulation of a reflectance map when varying the PhC radius. Other parameters are *a*_PhC_ = 1309 nm, *d*_PhC_ = 73 nm, vacuum gap 14.8 μm, and the GaAs substrate is a semi-infinite layer.

This interpretation is supported by rigorous coupled wave analysis (RCWA) simulations of our system^31,47^ (for parameters see Supporting Information). Figure 5b shows a simulated reflectance map when varying the PhC radius. We observe pronounced dips in reflectivity when hitting the cavity resonance condition. The simulated free spectral range is about 75 nm, which is close to the expected value given by the gap and noting that the PhC additionally modifies the effective cavity length.^48^ When decreasing *r*_PhC_, the cavity dip shifts to longer wavelengths implying an increased cavity length. This effect is also seen in the measurements, Figure 5a. Another dip occurs in the reflectance map, which originates from the coupling of focused light into a guided resonance of the PhC.^31,49^

To conclude, we have demonstrated that trampoline-shaped resonators in tensile-strained 73 nm thick InGaP exhibit mechanical quality factors surpassing 10^7^ at room temperature at pressures of 8 × 10^–6^ mbar, resulting in a *Q*·*f* product of 7 × 10^11^ Hz. An enhancement by a factor of 10 would place the presented InGaP trampoline mechanical resonators in the regime of quantum optomechanics at room temperature.^8^ The trampoline resonator was patterned with a PhC to engineer its out-of-plane reflectivity. We observed that the intrinsic mechanical quality factor of the InGaP mechanical resonators decreased over time. This undesired effect should receive future attention and may require surface passivation techniques.^46^ Once this issue is solved, mechanical dissipation in InGaP resonators can be further reduced by a simple increase of the tether length of the trampoline^8,9^ or by applying more sophisticated methods such as hierarchical clamping structures,^27^ machine-learning supported engineering of mechanical dissipation,^11,12^ quasi-phononic band gaps,^4^ or density phononic crystal engineering.^50^ Notably, the InGaP mechanical device layers can be incorporated in (Al,Ga)As heterostructures via epitaxial layer growth. This approach would allow the realization of integrated free-space cavity optomechanical systems in a crystalline material platform (see Supporting Information). Such compact optomechanical systems could implement bound-states in the continuum-based optomechanics,^51^ multielement,^52^ or hybrid optomechanical systems^53^ on a chip.

The data used in this work can be found in the open-access Zenodo database: 10.5281/zenodo.7441332.^54^

## Supporting Information Available

Details on fabrication, material properties, mechanical damping, parameters, and applications of the demonstrated nanomechanical resonators (PDF)
