# Supplementary material for: High-Q Trampoline Resonators from Strained Crystalline InGaP for Integrated Free-Space Optomechanics
Source: Nano Lett. 2023 May 26;23(11):5076–82. doi: 10.1021/acs.nanolett.3c00996 (PMC10273471; doi:10.1021/acs.nanolett.3c00996)
Supplement: Supplementary file 1 — nl3c00996_si_001.pdf [file nl3c00996_si_001.pdf]

Supplemental Material for: High-Q trampoline resonators from strained crystalline InGaP for integrated free-space optomechanics

Sushanth Kini Manjeshwar,[1] Anastasiia Ciers,[1] Fia Hellman,[1] Jürgen Bläsing,[2] André Strittmatter,[2] and Witlef Wieczorek[1,*]

[1] *Department of Microtechnology and Nanoscience (MC2), Chalmers University of Technology, SE-412 96 Gothenburg, Sweden*
[2] *Institute of Physics, Otto von Guericke Universität Magdeburg, 39106 Magdeburg, Germany*

(Dated: May 18, 2023)

CONTENTS

S1. Fabrication and InGaP material properties 1
A. Fabrication 1
B. Critical thickness 1
C. Anisotropic elasticity 1
D. Yield strength 3
E. Optical properties 3

S2. Mechanical damping 3
A. Clamping loss 4
B. Gas damping 4
C. Thermoelastic damping 4
D. Dilution factor in FEM 5

S3. Experimental parameters 6

S4. Applications 6

References 7

S1. FABRICATION AND INGAP MATERIAL PROPERTIES

A. Fabrication

Fig. S1 shows an SEM image of a fabricated and suspended InGaP trampoline. One can clearly see that the etched GaAs substrate surface under the trampoline is elevated and uneven. The unevenness will contribute to scattering loss of the optical cavity mode that forms between the suspended PhC trampoline and the substrate surface. The reason for this unevenness comes from the wet etch process. The PhC pattern determines the height profile of the "imprinted" surface onto the substrate. Basically, the wet etch process is faster on the large open areas outside of the trampoline pad than through the small diameter PhC holes. As a result, the material below the trampoline is initially etched slower than the material outside of the pad. At the end of the wet etch process, one obtains an elevated height profile mimicking the shape of the trampoline on the substrate surface.

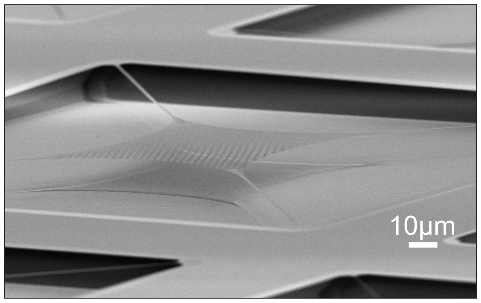

FIG. S1. Tilted SEM image of a PhC trampoline resonator. The etched GaAs substrate under the trampoline is clearly seen to exhibit an elevated and uneven surface with a height profile mimicking the PhC trampoline.

B. Critical thickness

Fig. S2 shows the critical thickness of $In_{1-x}Ga_xP$ on GaAs using the People and Bean model [1]. We observe that the thickness of $In_{0.43}Ga_{0.57}P$ from our work is well below the critical thickness criterion for the corresponding gallium content and hence the device layer does not relax to its native lattice constant and will therefore be tensile strained.

C. Anisotropic elasticity

The general linear relation between the components of the stress tensor σ_{ij} and strain tensor ϵ_{kl} is given by the fourth order stiffness tensor C_{ijkl} as

$$\sigma_{ij} = C_{ijkl}\epsilon_{kl}. \qquad \text{(S1)}$$

As InGaP has a zincblende crystal structure with a cubic symmetry, one can relate stress and strain with a

* witlef.wieczorek@chalmers.se

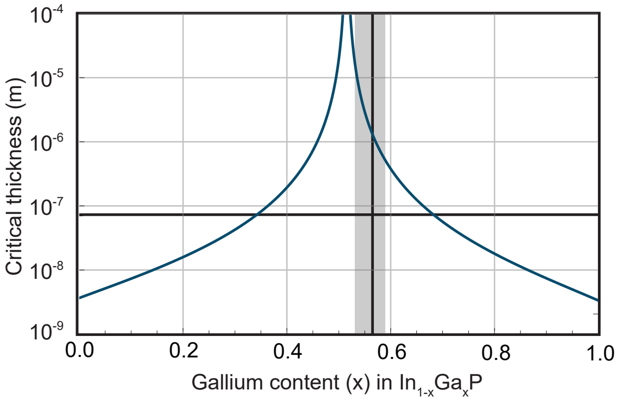

FIG. S2. Critical thickness of an $In_{1-x}Ga_xP$ layer grown on GaAs calculated using the People and Bean model [1]. The horizontal line represents the thickness of InGaP in this work, (73 nm). The gray area represents the range of gallium content estimated via X-ray diffraction and the vertical line represents the content that we use in this work ($x = 0.5658$).

reduced number of independent components by

$$\begin{pmatrix}\sigma_{xx}\\ \sigma_{yy}\\ \sigma_{zz}\\ \sigma_{yz}\\ \sigma_{xz}\\ \sigma_{xy}\end{pmatrix} = \begin{pmatrix} c_{11} & c_{12} & c_{12} & 0 & 0 & 0\\ c_{12} & c_{11} & c_{12} & 0 & 0 & 0\\ c_{12} & c_{12} & c_{11} & 0 & 0 & 0\\ 0 & 0 & 0 & c_{44} & 0 & 0\\ 0 & 0 & 0 & 0 & c_{44} & 0\\ 0 & 0 & 0 & 0 & 0 & c_{44}\end{pmatrix}\begin{pmatrix}\epsilon_{xx}\\ \epsilon_{yy}\\ \epsilon_{zz}\\ \epsilon_{yz}\\ \epsilon_{xz}\\ \epsilon_{xy}\end{pmatrix}, \quad \text{(S2)}$$

where x, y, z denote the crystal directions $[1\,0\,0]$, $[0\,1\,0]$, and $[0\,0\,1]$, respectively. The elastic constants of $In_{1-x}Ga_xP$, $c_{11}(x), c_{12}(x), c_{44}(x)$ [2], are listed in Tab. S1. The simplified matrix for C from Eq. S2 can be expressed in terms of material constants ($\nu_{ij} = -\epsilon_{jj}/\epsilon_{ii}, E_i = \sigma_{ii}/\epsilon_{ii}, G_{ij} = \sigma_{ij}/\epsilon_{ij}$) [3] as

$$C = \begin{pmatrix} \frac{1-\nu_{yz}\nu_{zy}}{E_yE_z\Delta} & \frac{\nu_{yx}+\nu_{yz}\nu_{zy}}{E_yE_z\Delta} & \frac{\nu_{zx}+\nu_{yz}\nu_{zy}}{E_yE_z\Delta} & 0 & 0 & 0\\ \frac{\nu_{xy}+\nu_{xz}\nu_{zy}}{E_yE_x\Delta} & \frac{1-\nu_{yx}\nu_{xz}}{E_yE_x\Delta} & \frac{\nu_{zy}+\nu_{yx}\nu_{xy}}{E_xE_z\Delta} & 0 & 0 & 0\\ \frac{\nu_{xz}+\nu_{xy}\nu_{yz}}{E_xE_y\Delta} & \frac{\nu_{yz}+\nu_{xz}\nu_{yx}}{E_xE_y\Delta} & \frac{1-\nu_{xy}\nu_{yx}}{E_xE_y\Delta} & 0 & 0 & 0\\ 0 & 0 & 0 & G_{yz} & 0 & 0\\ 0 & 0 & 0 & 0 & G_{zx} & 0\\ 0 & 0 & 0 & 0 & 0 & G_{xy}\end{pmatrix} \quad \text{(S3)}$$

where $\Delta = \frac{1-\nu_{xy}\nu_{yx}-\nu_{yz}\nu_{zy}-\nu_{zx}\nu_{zx}-2\nu_{xy}\nu_{yz}\nu_{xz}}{E_xE_yE_z}$.

The inverse of the stiffness matrix is the compliance matrix and relates strain to stress, $\epsilon_{ij} = S_{ijkl}\sigma_{kl}$, via

$$S = \begin{pmatrix} \frac{1}{E_x} & -\frac{\nu_{yx}}{E_y} & -\frac{\nu_{zx}}{E_z} & 0 & 0 & 0\\ -\frac{\nu_{xy}}{E_x} & \frac{1}{E_y} & -\frac{\nu_{zy}}{E_z} & 0 & 0 & 0\\ -\frac{\nu_{xz}}{E_x} & -\frac{\nu_{yz}}{E_y} & \frac{1}{E_z} & 0 & 0 & 0\\ 0 & 0 & 0 & \frac{1}{G_{yz}} & 0 & 0\\ 0 & 0 & 0 & 0 & \frac{1}{G_{zx}} & 0\\ 0 & 0 & 0 & 0 & 0 & \frac{1}{G_{xy}}\end{pmatrix} \quad \text{(S4)}$$

Thus, one can easily obtain Young's modulus in the x direction as $E_x = 1/s_{11}$. By rotating the matrices around the $[0\,0\,1]$-direction [4], Young's modulus in the $(0\,0\,1)$ plane can be obtained as [5]

$$E(x,\theta) = \\ = \frac{8\left[c_{11}(x)-c_{12}(x)\right]\left[c_{11}(x)+2c_{12}(x)\right]c_{44}(x)}{c_{11}^2(x)-2c_{12}(x)\left[c_{12}(x)-2c_{44}(x)\right]+c_{11}(x)\left[c_{12}(x)+6c_{44}(x)\right]+\left[c_{11}(x)+2c_{12}(x)\right]\left[c_{11}(x)-c_{12}(x)-2c_{44}(x)\right]\cos(4\theta)}. \quad \text{(S5)}$$

For a gallium content of $x = 0.5658$, $E(x, \theta)$ is plotted in Fig. S3(a).

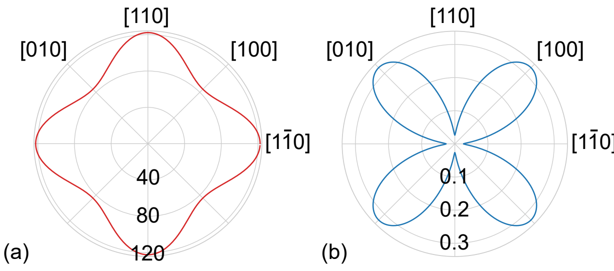

FIG. S3. Theoretical values for (a) Young's modulus (in GPa) and (b) Poisson's ratio.

As we are interested in the effect of the stress component along the string, we consider Poisson's ratio $\nu = \nu_{xy}$. This value can be obtained in the $(0\,0\,1)$ plane from the transformed compliance matrix as $\nu = -s_{21}/s_{11}$. The resulting Poisson's ratio is shown in Fig. S3(b).

We assume that the as-grown strain along 0° and 90° is the same, i.e., $\epsilon_{xx} = \epsilon_{yy} = \epsilon$. Then, the as-grown stress of $In_{1-x}Ga_xP$ for $x = 0.5658$ is $\sigma_{\text{grown}} = \sigma_{xx} = E_x\epsilon/(1-\nu)$. We obtain 472 MPa for the 0° direction.

The released stress in the string resonators can be obtained by taking into account Poisson's ratio, $\sigma_{\text{grown}}(1-\nu)$, resulting in 460 MPa and 319 MPa for a string oriented along 0° and 45°, respectively, see Fig. S4.

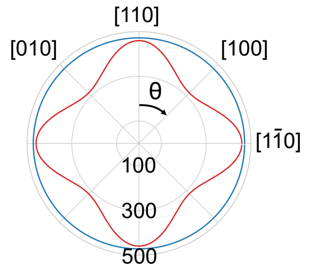

FIG. S4. As-grown stress, σ_{grown}, is in blue solid line, and released stress is in red (in MPa).

D. Yield strength

We fabricated nanostring resonators that are attached to the substrate with a tapered clamp of varying ratio $r = w_{\text{clamp}}/w_{\text{string}}$, where w_{clamp} is the width of the string at the clamping point and w_{string} is the width at the center of the string, see Fig. S5(a). The width at the clamping point determines the strain redistribution along the string resonator. The strain in the clamping region can be obtained from the strain-width relation as $\epsilon_{\text{clamp}} = \epsilon_{r=1} \cdot w_{\text{string}}/w_{\text{clamp}} = \epsilon_{r=1}/r$ [6]. As stress is proportional to strain, the stress at the clamping region is enhanced for $r < 1$ [Fig. S5(b)] and an increase above the yield stress of the material results in the fracturing of the beam. The corresponding yield stress is (for details see Ref. [6])

$$\sigma_{\text{yield}} = \sigma_{r=1}/r_{\text{yield}}. \tag{S6}$$

We fabricated 15 arrays of 40 µm-long and 2 µm-wide string resonators with r varying from 0.001 to 1 along the three aforementioned crystal directions. We observe that strings oriented along 0° [Fig. S5(c)], 45°, and 90° break for r_{yield} of (0.062 ± 0.005), (0.061 ± 0.007), and (0.067 ± 0.005), respectively. We determined $\sigma_{r=1}$ from a measurement of the fundamental mode frequency of the $r = 1$ resonator. We obtain yield stresses σ_{yield} using Eq. S6 of (5.5 ± 0.8) GPa, (3.3 ± 0.5) GPa, and (3.7 ± 0.5) GPa along 0°, 45°, and 90°, respectively.

To verify the analytic model for determining the yield strength, we perform independent FEM simulations of the corresponding tapered geometries [Fig. S5(b)] (parameters see Appendix [7]). Using r_{yield}, FEM simulations predict yield stresses of $\sigma_{\text{yield}}(0°, 90°) \approx 4.14$ GPa, $\sigma_{\text{yield}}(45°) \approx 3.2$ GPa and yield strain of ≈ 0.029, in reasonable agreement with the experimental values.

E. Optical properties

We measured the refractive index and absorption coefficient of the 73 nm-thick $In_{0.43}Ga_{0.57}P$ layer using an ellipsometer. The results are shown in Fig. S6 and resemble the values found in the literature for the wavelength range of interest in our work [8].

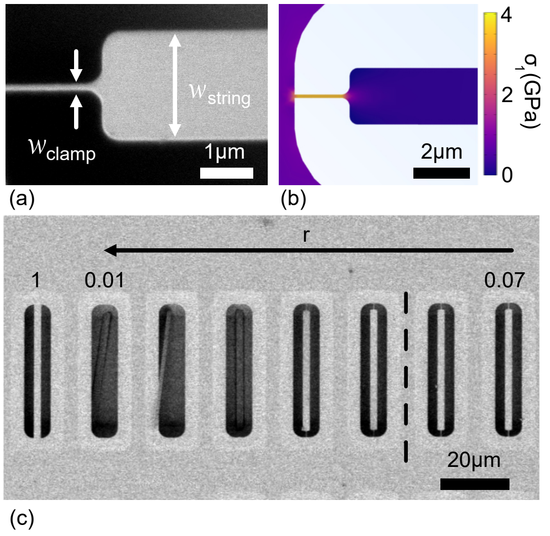

FIG. S5. Determination of the yield strength of the 73 nm-thick InGaP layer using tapered string resonators. (a) SEM image of the tapered clamp region. (b) FEM simulation of the first principal stress, σ_1, of the tapered string. (c) SEM image of tapered string resonators oriented along 0°. Resonators of $r < 0.06$ (dashed line) fracture.

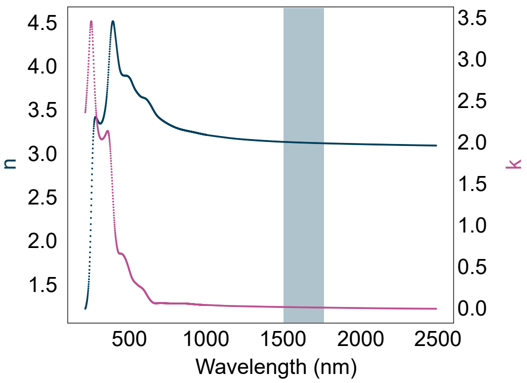

FIG. S6. Refractive index n and extinction coefficient k for the 73 nm-thick InGaP film determined from ellipsometer measurements. The refractive index n is in agreement with Ref. [8].

S2. MECHANICAL DAMPING

The total mechanical quality factor of a mechanical resonator is given by [9, 10]

$$Q^{-1} = Q_{\text{int}}^{-1} + Q_{\text{ext}}^{-1} = \sum_i \left(Q_{\text{int}}^i\right)^{-1} + \sum_j \left(Q_{\text{ext}}^j\right)^{-1}, \tag{S7}$$

where Q_{int}^i originates, amongst others, from surface loss, thermoelastic damping, or loss processes related to ma-

terial defects, and $Q^i_{\rm ext}$ from, amongst others, gas damping or clamping loss. For an in-depth discussion, see Refs. [9, 10]. In the following, we analyze mechanical loss processes relevant to this work.

A. Clamping loss

Clamping loss is determined by the geometry of the clamping of the mechanical resonator to the substrate. Clamping results in the transfer of acoustic energy from the mechanical resonator to its environment. In the following, we will estimate the clamping loss for string-type resonators and compare it to the one of a cantilever and a fully clamped square membrane which would give the lower and upper bounds for clamping loss, respectively. Further, we will examine the clamping loss of a fully clamped membrane to obtain an upper limit to the clamping loss of a trampoline resonator, which we also independently simulated with FEM.

For string resonators in this work, we can write the total mechanical quality factor as

$$Q^{-1}_{\rm total} = \frac{1}{D_n \cdot Q_{\rm int}} + \frac{1}{Q_{\rm clamp,s}}. \tag{S8}$$

Similar to Ref. [11], we assume $Q_{\rm clamp,s} = \eta L/h$, where η is a free parameter. For the string resonators in our work and using Eq. S8, we obtain $Q_{\rm clamp,s} \sim 10^{12}$.

We can compare the clamping loss of the doubly-clamped string resonators to the one of singly-clamped cantilevers, where the latter will give an upper bound on $Q_{\rm clamp}$ for string resonators. The clamping loss of a cantilever of thickness h, width w and length L is given by [10]

$$Q^{-1}_{\rm clamp,c} = 0.31 \frac{w}{L}\left(\frac{h}{L}\right)^4, \tag{S9}$$

for the case where the thickness of the substrate is comparable to the wavelength of the acoustic wave. The fundamental mode of a 155 µm-long and 2 µm-wide cantilever has $Q_{\rm clamp,c} = 5 \cdot 10^{15}$, which is larger than $Q_{\rm clamp,s}$ of a string with the same dimensions.

Similarly, we obtain the lower bound on $Q_{\rm clamp,s}$ by examining a worst-case scenario of a fully clamped membrane. The clamping loss-limited Q factor of a tensile-stressed square membrane clamped to a thick substrate is given by [10, 12]

$$Q_{\rm clamp,m} \approx \frac{3}{2}\sqrt{\frac{\rho_{\rm InGaP}}{\rho_{\rm GaAs}}}\left(\frac{E_{\rm GaAs}}{\sigma}\right)^{3/2} \frac{n^2 m^2}{(n^2+m^2)^{3/2}}\frac{L}{h}, \tag{S10}$$

where h is the thickness of the membrane, L the length of one of its sides, $\rho_{\rm InGaP}$ and $\rho_{\rm GaAs}$ are the densities of the membrane and the substrate, respectively, $E_{\rm GaAs}$ is Young's modulus of the GaAs substrate, n and m are the eigenmode numbers of the mechanical mode. For a $155{\times}155\,\mu\mathrm{m}^2$ square membrane with a uniform tensile stress of 415 MPa, $Q_{\rm clamp,m}$ for the fundamental mode is about $7 \cdot 10^6$. As we observe Q factors of string resonators $\leq 3 \cdot 10^6$, we conclude that they are not limited by clamping loss.

Now, we estimate a lower bound on $Q_{\rm clamp}$ for the trampoline resonators by using Eq. S10. For a $1130{\times}1130\,\mu\mathrm{m}^2$ square membrane (corresponding to a trampoline with tether length of 750 µm and central pad size of 100 µm) with uniform tensile stress of 415 MPa, $Q_{\rm clamp,m}$ for the fundamental mode is about $5 \cdot 10^7$. This value yields a lower limit for the clamping loss-related Q factor of trampoline resonators. We also performed FEM simulations to estimate $Q_{\rm clamp,m}$ for the trampoline resonators using perfectly matched layers [13]. We find that $Q_{\rm clamp} > 10^8$ [14]. Therefore, we consider that the micromechanical resonators of this work are not clamping-loss limited.

B. Gas damping

Gas damping is another extrinsic mechanical damping process [9]. For a mechanical resonator, such as a string- or membrane-type resonator, oscillating at frequency Ω_m at low pressures P, i.e., in the ballistic regime, where the mean free path of the gas molecules is larger than the dimensions of the mechanical resonator, $Q_{\rm gas}$ is given as [15]

$$Q^{-1}_{\rm gas} = 4\sqrt{\frac{2}{\pi}}\frac{1}{\rho h \Omega_m} P \sqrt{\frac{M}{RT}}, \tag{S11}$$

where M is the molecular mass of the gas molecules, R is the molar gas constant, and T is the temperature of the gas.

Fig. S7 shows pressure-dependent measurements for InGaP string resonators of length 155 µm and width 2 µm oriented along 0°. For pressures $P > 10^{-2}$ mbar, the Q factor of the strings is limited by gas damping, while for pressures $P < 10^{-2}$ mbar, Q is constant, indicating another limiting damping mechanism.

C. Thermoelastic damping

Thermoelastic damping (TED) is related to the irreversible conversion of mechanical energy into heat [9]. During the oscillation of the mechanical resonator, one of its sides is compressed, while the opposite side is stretched. This deformation produces a temperature gradient and, thus, an irreversible transport of heat, in case the thermal expansion coefficient α is non-zero.

For a thin beam of length L, thickness h, and width w at temperature T the quality factor limited by TED, $Q_{\rm TED}$, is given by [16]

$$Q^{-1}_{\rm TED,\,L\text{-}R} = \frac{\alpha^2 E T}{C_p}\left(\frac{6}{\xi^2} - \frac{6}{\xi^3}\frac{\sinh\xi + \sin\xi}{\cosh\xi + \cos\xi}\right), \tag{S12}$$

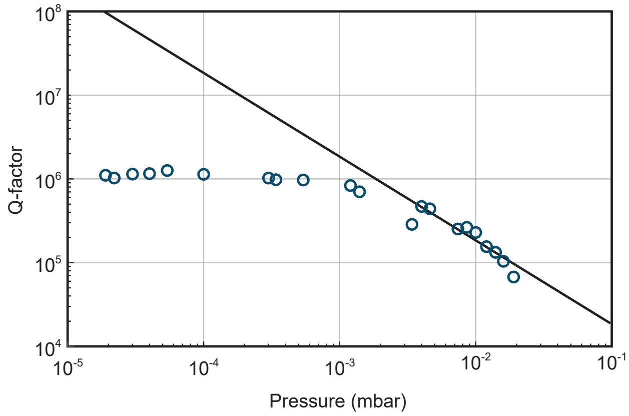

FIG. S7. Pressure-dependent measurement of the Q factor of InGaP string resonators. The predicted gas damping from Eq. S11 is shown as solid line.

where $\xi = h\sqrt{\frac{\omega_0}{2\chi}}$ is a dimensionless variable, with ω_0 the isothermal value of the eigenfrequency ω, $\chi = \kappa/\rho C_p$ the thermal diffusivity, C_p the heat capacity per unit volume at constant pressure, and κ the thermal conductivity. At room temperature, the linear thermal expansion coefficient is $\alpha \approx 4.28 \times 10^{-6}$ 1/K, based on the experimental values for InP and GaP [17]. For this isotropic model, we set Young's modulus to $E = 85\,\text{GPa}$. Based on Eq. S12, we obtain the straight line in Fig. S8 for $Q_{\text{TED, L-R}}$. We independently performed FEM simulations of beams with the same dimensions and material parameters and obtain very similar results (see Fig. S8).

As our InGaP resonators are strained we need to modify the thermal relaxation time and the oscillation frequency in the model above [18]. Following the derivation from Ref. [19] the pre-stress is included via an additional axial force $\sigma_0 = \frac{F}{wh}$, which leads to Q_{TED} for strained beams

$$Q_{\text{TED}}^{-1} = \frac{1}{1 + a\frac{F}{F_{\text{cr}}}} \cdot Q_{\text{TED, L-R}}^{-1}(\xi), \tag{S13}$$

where $F_{\text{cr}} = \pi EI/L^2$, $a = 0.97$ is a factor that depends on the boundary condition [20], I is the moment of inertia, and ξ gets modified by inserting $\omega_0 = \frac{\pi}{L^2}\sqrt{\frac{EI}{\rho wh}}\sqrt{\pi^2 + \frac{FL^2}{EI}}$.

For a stress of $\sigma_0 = 300\,\text{MPa}$, Q_{TED} of a strained beam is shown as the dashed line in Fig. S8 and considerably larger than $Q_{\text{TED, L-R}}$. We also simulated such stressed beams in FEM and obtain a good agreement with the analytical estimate Eq. S13 (see Fig. S8).

Estimating TED of the trampoline patterned with a PhC should take into account the periodic perforation of the material, which leads to an effective medium with a weaker elasticity matrix [21] and periodic stress redistribution. The periodic strain variation results in a temperature gradient around the PhC hole edges and, therefore, in an increase of TED loss [22].

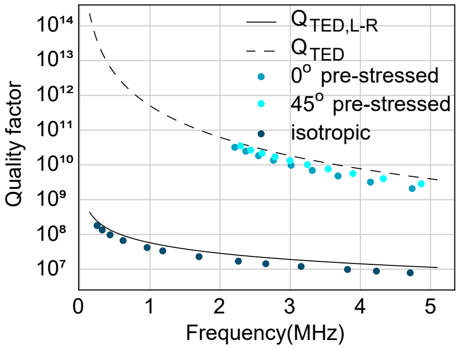

FIG. S8. TED loss in beams. Analytical and FEM models of Q_{TED} for 2 µm-wide, 73 nm-thick InGaP beams. Solid line is Eq. S12, dashed line is Eq. S13 and dots are FEM results.

To get an estimate of Q_{TED} for a PhC trampoline resonator, we performed FEM simulations (using $r_{\text{PhC}} = 552\,\text{nm}$, $a_{\text{PhC}} = 1323\,\text{nm}$) and obtained $Q_{\text{TED}} \approx 10^8$ for the fundamental mode at a frequency of $\Omega_m/2\pi = 163\,\text{kHz}$, which is higher than the Q we observed in the experiment.

D. Dilution factor in FEM

Q_{int} can be generally written as the ratio of the stored versus the lost energy over one cycle of oscillation [10]:

$$Q_{\text{int}} = 2\pi\frac{W_{\text{elongation}} + W_{\text{bending}}}{\Delta W_{\text{elongation}} + \Delta W_{\text{bending}}}, \tag{S14}$$

where $W_{\text{elongation}}$ and W_{bending} are the potential energy stored in the elongation and bending of the resonator, respectively. Similarly, $\Delta W_{\text{elongation}}$ and $\Delta W_{\text{bending}}$ are the corresponding loss processes.

The quality factor can be increased by diluting the intrinsic material friction. This can be achieved by utilizing materials with high intrinsic tensile stress, which introduces W_{tensile} as an additional contribution to the potential energy. The increased quality factor Q_{D} is then given by [6, 23]

$$Q_{\text{D}} = D \cdot Q_{\text{int}}, \tag{S15}$$

where D is the dilution factor that depends on the pre-strain of the material, resonator geometry, and the displacement mode profile. The dilution factor is in general given by [23]

$$D = 1 + \frac{W_{\text{tensile}}}{W_{\text{elongation}} + W_{\text{bending}}}. \tag{S16}$$

We see that by increasing the elastic energy W_{tensile}, the mechanical quality factor of the resonator can be increased to a considerable extent, with record values of $Q > 10^{10}$ in highly tensile-strained crystalline silicon [24].

For thin two-dimensional membranes (i.e., membranes with a large length-to-thickness ratio) with the out-of-plane displacement $u_z = w(x,y)$, the dilution factor can be calculated using FEM. In the case of $D_Q \gg 1$, the first term in Eq. S16 can be neglected. Then, the dissipation dilution factor can be calculated by integrating over the entire membrane area as [25]

$$D_Q = \frac{\rho h \Omega_m^2}{D_p} \frac{\iint w(x,y)^2 dxdy}{\iint \left\{ \left(\frac{\partial^2 w(x,y)}{\partial x^2} + \frac{\partial^2 w(x,y)}{\partial y^2} \right)^2 + 2(1-\nu) \left[\left(\frac{\partial^2 w(x,y)}{\partial x \partial y} \right)^2 - \frac{\partial^2 w(x,y)}{\partial x^2} \frac{\partial^2 w(x,y)}{\partial y^2} \right] \right\} dxdy} \tag{S17}$$

where $D_p = Eh^3/[12(1-\nu^2)]$ is the flexural rigidity of the material and ν is Poisson's ratio. The numerator yields the kinetic energy of the membrane and the denominator the energy stored in the membrane bending [26].

S3. EXPERIMENTAL PARAMETERS

Tab. S1 summarizes the material and device parameters of this work.

Parameter	Symbol	Value
**Material properties**		
Ga content	x	0.5658
Density (kg/m^3)	ρ	$(4.81 - 0.67x) \cdot 10^3$
Thickness (nm)	h, d_{PhC}	73
Stress (MPa)	$\sigma(0°)$	467.7 ± 7.1
	$\sigma(45°)$	313.3 ± 5.4
	$\sigma(90°)$	374.9 ± 16.4
Elastic constants (Pa)	c_{11}	$(10.11 + 3.94x) \cdot 10^{10}$
at 300 K	c_{12}	$(5.61 + 0.59x) \cdot 10^{10}$
	c_{44}	$(4.56 + 2.47x) \cdot 10^{10}$
Lattice constant [2] (Å)		
$In_{1-x}Ga_xP$	$a_{In_{1-x}Ga_xP}$	$(5.8687 - 0.4182x)$
GaAs	a_{GaAs}	5.65325
Thermal diffusivity (W/K)	κ	66
Heat capacity (J/kgK)	C_p	380
Linear thermal expansion coefficient at RT (1/K)	α	$4.28 \cdot 10^{-6}$
**Optical properties**		
Refractive index	n	3.15
Gap (µm)	L_{gap}	14.8
**Mechanical properties**		
Effective mass (ng)	m_{eff}	9.3

TABLE S1. Parameters used in FEM and RCWA simulations.

S4. APPLICATIONS

We have demonstrated InGaP trampoline mechanical resonators with a quality factor of 10^7 at room temperature with a resonance frequency of 38 kHz, resulting in a $Q \cdot f_m$ product of $7 \cdot 10^{11}$ Hz.

In the following, we discuss future device improvements and mention applications, where improved InGaP trampoline resonators are particularly fitting. We focus our discussion on InGaP mechanical resonators that offer a central area amenable to patterning with a photonic crystal (PhC). We deliberately choose the use of a suspended PhC as engineering the reflectance of the mechanical resonator via the PhC enables efficient transduction of mechanical displacement to out-of-plane light fields, as was recently demonstrated [27]. Furthermore, the (Al,Ga)As combined with InGaP material platform provides the opportunity to realize integrated free-space optomechanical microcavities [28]. Another interesting avenue would be to pattern phononic-shielded string-like resonators in InGaP [29, 30], which we will not discuss in the following.

a. Improvements of mechanical performance Figures of merit in nanomechanics are the mechanical frequency f_m, the mechanical Q factor, and the effective mass m_{eff} of the resonator. An increase in Q and f_m can be achieved by increasing the Ga content of InGaP to obtain a tensile stress of up to 1 GPa. This is a factor of two higher than the stress in the current samples and would translate into a factor of about $\sqrt{2}$ enhancement of f_m. In general, the tensile strain of the InGaP layer allows employing more advanced strain engineering techniques than we have currently used. This comprises optimized trampoline designs (with Q factors up to 10^8 at $f_m \sim 150$ kHz in SiN [31]), hierarchical clamping (Q factor larger than 10^8 at $f_m \sim 100$ kHz in SiN [29]), or phononic-shield membranes (Q-factor values larger than 10^8 at $f_m \sim 700$ kHz in SiN [32]). Furthermore, decreasing the thickness of the InGaP layer by a factor of two to about 35 nm, would lead to a further increase of Q, with a concomitant decrease of f_m.

Overall, we can expect to reach Q factors of InGaP trampoline-like resonators larger than 10^8 at frequencies of between 50 to 100 kHz with an effective mass between 1 to 10 ng. Furthermore, we expect that operating InGaP resonators at low temperatures will result in a further enhancement of Q, as observed in other crystalline materials such as diamond [33] or silicon [24, 33], with an increase of up to a factor of five.

b. Force sensing For our current InGaP trampolines, we estimate a thermal noise limited force sensitivity of $F_{\mathrm{th}} \approx 50\,\mathrm{aN}/\sqrt{\mathrm{Hz}}$ (for an effective mass of 10 ng). Improved InGaP trampoline resonators with an effective mass of 1 ng at a frequency of 50 kHz and a Q of 10^8 would yield a thermal noise limited force sensitivity at room temperature of $7\,\mathrm{aN}/\sqrt{\mathrm{Hz}}$, which would be better than the one achieved with SiN trampolines ($19.5\,\mathrm{aN}/\sqrt{\mathrm{Hz}}$ [31]) or phononic-shield membranes ($37\,\mathrm{aN}/\sqrt{\mathrm{Hz}}$ [32, 34]) and similar to other mechanical resonator geometries designed for force sensing [35–37]. When operating the devices at lower temperatures, e.g., at 4 K, we estimate $0.37\,\mathrm{aN}/\sqrt{\mathrm{Hz}}$ when also assuming a Q increase by a factor of 5 upon cooling. This value is close to the sensitivity required to detect the magnetic moment of a single proton [38].

c. Quantum optomechanics at room temperature Improved InGaP trampoline resonators with a frequency of 100 kHz and a Q of 10^8 would yield a $Q{\cdot}f \sim 10{\cdot}10^{12}$ Hz, which places such devices in the regime of quantum optomechanics at room temperature. As the trampoline can be patterned with a high-reflectivity PhC, it can be directly used as a mirror in an optical cavity, which enables feedback cooling of a trampoline mechanical mode to the ground state [39, 40].

d. Optomechanical microcavity We have recently demonstrated an optomechanical microcavity based on an (Al,Ga)As heterostructure [28], which accesses the regime of ultra-strong optomechanical coupling, with $\omega_m \sim 0.3 g_0$. However, these experiments were limited by the large optical loss rate of hundreds of GHz, placing the system deep in the bad cavity regime, and the low mechanical $Q \sim 10^4$ of the suspended GaAs-based mechanical resonator.

Using InGaP trampolines for such an integrated optomechanical microcavity would allow reaching Q factors that are four orders of magnitude larger compared to GaAs-based mechanical resonators. Furthermore, the strained InGaP layer enables patterning of a PhC on a trampoline with a sufficiently large central pad of $250\,\mu\mathrm{m}\times 250\,\mu\mathrm{m}$, such that diffraction loss and collimation or finite size effects [41] of the PhC when illuminated with a $50\,\mu\mathrm{m}$ waist are estimated to be negligible. A reflectivity above 0.9998 has been recently demonstrated with a similarly sized suspended PhC membrane at telecom wavelength [27], yielding a Finesse $\mathcal{F} > 3 \cdot 10^4$.

An envisioned InGaP-based optomechanical microcavity with a length $L_c \sim \lambda/2$ at $\lambda_0 = 1550\,\mathrm{nm}$, would yield an optical loss rate $\kappa = \pi c/(2 L_c \mathcal{F}) < 2\pi \cdot 3\,\mathrm{GHz}$. Thanks to the short length of $\lambda_0/2$ of the microcavity a single-photon coupling strength $g_0 \sim 2\pi \cdot 0.9\,\mathrm{MHz}$ is within reach. This optomechanical microcavity would be placed in the ultra-strong coupling ($g_0/\omega_m \sim 9$ with $\omega_m = 2\pi \cdot 100\,\mathrm{kHz}$) and bad cavity regime ($g_0/\kappa \sim 3 \cdot 10^{-4}$). With a Q of 10^8 ($5 \cdot 10^8$) at room temperature (4 K), a single-photon cooperativity $C_0 = (g_0)^2/\kappa\gamma n_{th}$ of $4 \cdot 10^{-3}$ (1.6) can be achieved, with the phonon occupation $n_{th} = k_B T/\hbar\omega_m$. Such a system will allow studying novel optomechanical effects in the ultra-strong coupling regime [42] and realization of mechanical squeezing as recently proposed in Ref. [43].

An integrated optomechanical microcavity may also be able to access the single-photon strong coupling regime, which is so far elusive in nano- or micromechanics. One approach relies on multi-element optomechanics [44], which aims at increasing g_0 by virtue of collective mechanical effects between highly reflective mechanical elements placed within a cavity. We have taken steps in this direction in the (Al,Ga)As-based system by realizing two-element mechanics on a chip [28]. Using InGaP instead of GaAs-based mechanics would thereby allow reaching higher Q mechanical resonators. Further, a key challenge with this approach is to match the mechanical frequency of the resonators within their linewidth. As InGaP is a piezoelectric material, one can tune the mechanical frequency in-situ via application of a static voltage, which is a desired feature to realize the stringent requirements of Ref. [44].

Another approach to reach the single-photon strong coupling regime relies on minimizing the optical loss rate κ via the use of photonic bound states in the continuum [45]. A system composed of two InGaP-based suspended PhC mirrors at a well-defined spacing, e.g., defined via heterostructure growth, can realize such a photonic bound state in the continuum, where it has to be seen in the experiment how small κ can be realized given fabrication imperfections of the PhC and the material's absorption.

[1] R. People and J. C. Bean, Calculation of critical layer thickness versus lattice mismatch for GexSi1-x/Si strained-layer heterostructures, Applied Physics Letters **47**, 322 (1985).

[2] M. S. Shur, *Handbook Series on Semiconductor Parameters, Vol. 2: Ternary and Quaternary III-V Compounds* (World Scientific, 1996).

[3] M. A. Hopcroft, W. D. Nix, and T. W. Kenny, What is the young's modulus of silicon?, J. Microelectromechanical Systems **19**, 229 (2010).

[4] J. Wortman and R. Evans, Young's modulus, shear modulus, and poisson's ratio in silicon and germanium, Journal of applied physics **36**, 153 (1965).

[5] M. Bückle, V. C. Hauber, G. D. Cole, C. Gärtner, U. Zeimer, J. Grenzer, and E. M. Weig, Stress control of tensile-strained $\mathrm{In}_x\mathrm{Ga}_{1-x}\mathrm{P}$ nanomechanical string resonators, Applied Physics Letters **113**, 201903 (2018).

[6] S. A. Fedorov, N. J. Engelsen, A. H. Ghadimi, M. J. Bereyhi, R. Schilling, D. J. Wilson, and T. J. Kippenberg, Generalized dissipation dilution in strained mechanical resonators, Phys. Rev. B **99**, 054107 (2019).

[7] See supplemental material for detailed information on

fabrication, material properties, mechanical damping and parameters.
[8] S. Adachi, Refractive indices of iii–v compounds: Key properties of ingaasp relevant to device design, Journal of Applied Physics **53**, 5863 (1982).
[9] M. Imboden and P. Mohanty, Dissipation in nanoelectromechanical systems, Physics Reports **534**, 89 (2014).
[10] S. Schmid, L. G. Villanueva, and M. L. Roukes, *Fundamentals of nanomechanical resonators*, Vol. 49 (Springer, 2016).
[11] S. Schmid, K. D. Jensen, K. H. Nielsen, and A. Boisen, Damping mechanisms in high-Q micro and nanomechanical string resonators, Phys. Rev. B **84**, 165307 (2011).
[12] L. G. Villanueva and S. Schmid, Evidence of surface loss as ubiquitous limiting damping mechanism in sin micro- and nanomechanical resonators, Physical review letters **113**, 227201 (2014).
[13] E. Romero, V. M. Valenzuela, A. R. Kermany, L. Sementilli, F. Iacopi, and W. P. Bowen, Engineering the dissipation of crystalline micromechanical resonators, Physical Review Applied **13**, 044007 (2020).
[14] F. Hellman, *Tensile-strained micromechanical resonators made from crystalline InGaP with low mechanical dissipation and high optical reflectivity*, Master's thesis, Chalmers university of technology, Göteborg, Sweden (2022).
[15] S. S. Verbridge, R. Ilic, H. G. Craighead, and J. M. Parpia, Size and frequency dependent gas damping of nanomechanical resonators, Applied Physics Letters **93**, 013101 (2008).
[16] R. Lifshitz and M. L. Roukes, Thermoelastic damping in micro-and nanomechanical systems, Physical review B **61**, 5600 (2000).
[17] H.-M. Kagaya and T. Soma, Mode grüneisen parameters and thermal expansion of gap and inp, Solid state communications **58**, 479 (1986).
[18] C. Zener, Internal friction in solids ii. general theory of thermoelastic internal friction, Physical review **53**, 90 (1938).
[19] S. Kumar and M. Aman Haque, Stress-dependent thermal relaxation effects in micro-mechanical resonators, Acta mechanica **212**, 83 (2010).
[20] A. Bokaian, Natural frequencies of beams under tensile axial loads, Journal of sound and vibration **142**, 481 (1990).
[21] V. L. Rabinovich, R. K. Gupta, and S. D. Senturia, The effect of release-etch holes on the electromechanical behaviour of mems structures, in *Proceedings of International Solid State Sensors and Actuators Conference (Transducers' 97)*, Vol. 2 (IEEE, 1997) pp. 1125–1128.
[22] C. Tu and J. E. Lee, Study on thermoelastic dissipation in bulk mode resonators with etch holes, in *2012 7th IEEE International Conference on Nano/Micro Engineered and Molecular Systems (NEMS)* (IEEE, 2012) pp. 478–482.
[23] L. Sementilli, E. Romero, and W. P. Bowen, Nanomechanical dissipation and strain engineering, Advanced Functional Materials **32**, 2105247 (2022).
[24] A. Beccari, D. A. Visani, S. A. Fedorov, M. J. Bereyhi, V. Boureau, N. J. Engelsen, and T. J. Kippenberg, Strained crystalline nanomechanical resonators with quality factors above 10 billion, Nature Physics **18**, 436 (2022).
[25] S. Fedorov, *Mechanical resonators with high dissipation dilution in precision and quantum measurements*, Ph.D. thesis, EPFL, Lausanne (2020).
[26] L. D. Landau, E. M. Lifšic, E. M. Lifshitz, A. M. Kosevich, and L. P. Pitaevskii, *Theory of elasticity: volume 7*, Vol. 7 (Elsevier, 1986).
[27] F. Zhou, Y. Bao, J. J. Gorman, and J. Lawall, Cavity optomechanical bistability with an ultrahigh reflectivity photonic crystal membrane (2022), 2211.10485 [physics].
[28] S. Kini Manjeshwar, *Free-space cavity optomechanical systems on a chip with III-V heterostructures*, Ph.D. thesis, Chalmers University of Technology, Gothenburg, Sweden (2023).
[29] M. J. Bereyhi, A. Beccari, R. Groth, S. A. Fedorov, A. Arabmoheghi, T. J. Kippenberg, and N. J. Engelsen, Hierarchical tensile structures with ultralow mechanical dissipation, Nature Communications **13**, 1 (2022).
[30] M. J. Bereyhi, A. Arabmoheghi, A. Beccari, S. A. Fedorov, G. Huang, T. J. Kippenberg, and N. J. Engelsen, Perimeter modes of nanomechanical resonators exhibit quality factors exceeding 10^9 at room temperature, Phys. Rev. X **12**, 021036 (2022).
[31] R. A. Norte, J. P. Moura, and S. Gröblacher, Mechanical resonators for quantum optomechanics experiments at room temperature, Physical review letters **116**, 147202 (2016).
[32] Y. Tsaturyan, A. Barg, E. S. Polzik, and A. Schliesser, Ultracoherent nanomechanical resonators via soft clamping and dissipation dilution, Nature nanotechnology **12**, 776 (2017).
[33] Y. Tao, J. M. Boss, B. A. Moores, and C. L. Degen, Single-crystal diamond nanomechanical resonators with quality factors exceeding one million, Nature Communications **5**, 3638 (2014).
[34] D. Hälg, T. Gisler, Y. Tsaturyan, L. Catalini, U. Grob, M.-D. Krass, M. Héritier, H. Mattiat, A.-K. Thamm, R. Schirhagl, E. C. Langman, A. Schliesser, C. L. Degen, and A. Eichler, Membrane-Based Scanning Force Microscopy, Phys. Rev. Applied **15**, L021001 (2021).
[35] M. Héritier, A. Eichler, Y. Pan, U. Grob, I. Shorubalko, M. D. Krass, Y. Tao, and C. L. Degen, Nanoladder Cantilevers Made from Diamond and Silicon, Nano Lett. **18**, 1814 (2018).
[36] S. L. de Bonis, C. Urgell, W. Yang, C. Samanta, A. Noury, J. Vergara-Cruz, Q. Dong, Y. Jin, and A. Bachtold, Ultrasensitive Displacement Noise Measurement of Carbon Nanotube Mechanical Resonators, Nano Lett. **18**, 5324 (2018).
[37] P. Sahafi, W. Rose, A. Jordan, B. Yager, M. Piscitelli, and R. Budakian, Ultralow Dissipation Patterned Silicon Nanowire Arrays for Scanning Probe Microscopy, Nano Lett. **20**, 218 (2020).
[38] Y. Tao, A. Eichler, T. Holzherr, and C. L. Degen, Ultrasensitive mechanical detection of magnetic moment using a commercial disk drive write head, Nat Commun **7**, 12714 (2016).
[39] M. Rossi, D. Mason, J. Chen, Y. Tsaturyan, and A. Schliesser, Measurement-based quantum control of mechanical motion, Nature **563**, 53 (2018).
[40] L. Magrini, P. Rosenzweig, C. Bach, A. Deutschmann-Olek, S. G. Hofer, S. Hong, N. Kiesel, A. Kugi, and M. Aspelmeyer, Real-time optimal quantum control of mechanical motion at room temperature, Nature **595**, 373 (2021).
[41] C. Toft-Vandborg, A. Parthenopoulos, A. A. Darki, and A. Dantan, Collimation and finite-size effects in sus-

pended resonant guided-mode gratings, J. Opt. Soc. Am. A, JOSAA **38**, 1714 (2021).
[42] A. Frisk Kockum, A. Miranowicz, S. De Liberato, S. Savasta, and F. Nori, Ultrastrong coupling between light and matter, Nat Rev Phys **1**, 19 (2019).
[43] K. Kustura, C. Gonzalez-Ballestero, A. d. l. R. Sommer, N. Meyer, R. Quidant, and O. Romero-Isart, Mechanical Squeezing via Unstable Dynamics in a Microcavity, Phys. Rev. Lett. **128**, 143601 (2022).
[44] A. Xuereb, C. Genes, and A. Dantan, Strong Coupling and Long-Range Collective Interactions in Optomechanical Arrays, Phys. Rev. Lett. **109**, 223601 (2012).
[45] J. M. Fitzgerald, S. K. Manjeshwar, W. Wieczorek, and P. Tassin, Cavity optomechanics with photonic bound states in the continuum, Phys. Rev. Research **3**, 013131 (2021).
